# Elevated Plasma Corticosterone Decreases Yolk Testosterone and Progesterone in Chickens: Linking Maternal Stress and Hormone-Mediated Maternal Effects

**DOI:** 10.1371/journal.pone.0023824

**Published:** 2011-08-23

**Authors:** Rie Henriksen, Ton G. Groothuis, Sophie Rettenbacher

**Affiliations:** 1 Behavioural Biology, Institute of Behavioural Neuroscience, University of Groningen, Groningen, The Netherlands; 2 Biochemistry, University of Veterinary Medicine Vienna, Vienna, Austria; Pennsylvania State University, United States of America

## Abstract

Despite considerable research on hormone-mediated maternal effects in birds, the underlying physiology remains poorly understood. This study investigated a potential regulation mechanism for differential accumulation of gonadal hormones in bird eggs. Across vertebrates, glucocorticoids can suppress reproduction by downregulating gonadal hormones. Using the chicken as a model species, we therefore tested whether elevated levels of plasma corticosterone in female birds influence the production of gonadal steroids by the ovarian follicles and thus the amount of reproductive hormones in the egg yolk. Adult laying hens of two different strains (ISA brown and white Leghorn) were implanted subcutaneously with corticosterone pellets that elevated plasma corticosterone concentrations over a period of nine days. Steroid hormones were subsequently quantified in plasma and yolk. Corticosterone-implanted hens of both strains had lower plasma progesterone and testosterone levels and their yolks contained less progesterone and testosterone. The treatment also reduced egg and yolk mass. Plasma estrogen concentrations decreased in white Leghorns only whereas in both strains yolk estrogens were unaffected. Our results demonstrate for the first time that maternal plasma corticosterone levels influence reproductive hormone concentrations in the yolk. Maternal corticosterone could therefore mediate environmentally induced changes in yolk gonadal hormone concentrations. In addition, stressful situations experienced by the bird mother might affect the offspring via reduced amounts of reproductive hormones present in the egg as well as available nutrients for the embryo.

## Introduction

Birds have proven to be excellent models to study hormone-mediated maternal effects in an evolutionary framework [1]. Pioneered by Schwabl [2], research on avian yolk steroid hormones has quickly expanded and has revealed for example that concentrations of yolk hormones vary according to the prevailing environmental conditions experienced by the laying female, such as habitat quality in wild birds [1] or the housing conditions in domestic chickens [3]. Other studies found that the mothers' own body condition [4] and the quality of her partner [5] can affect the concentrations of yolk steroid hormones. Changes over the laying season, as maternal condition deteriorates, have also been discovered [1], [6]. Besides androgens, which are investigated in the majority of studies, also gestagens and estrogens have been measured in the yolk of various bird species [2], [7]–[10] and environmental cues perceived by the female bird also seem to influence their concentrations [3], [11], [12]. Since yolk hormones affect several fitness components of the embryo, nestling and juvenile stages, maternal manipulation of yolk hormones is regarded a strategy to maximize maternal fitness [1], [13], [14]. Although adaptive functional and evolutionary studies continue to proliferate rapidly, the underlying physiological mechanism that enables the female to regulate the steroid hormone content of her eggs remains unresolved, hampering progress in the field [15]. In mammals, glucocorticoids (GC) can influence the synthesis of gonadal steroids both by downregulation of the gonadotrophins [16], [17] and via direct effects on the testis and the ovaries [18]–[20]. Similarly, it has been shown for birds that concentrations of LH decrease due to corticosterone elevation [21], [22], but stress might also directly inhibit gonadal hormone production [23]. In the present study we therefore explored the potential role of GC on the synthesis of reproductive hormones and their accumulation in the egg. Our aim was to test whether corticosterone from the peripheral circulation influences gonadal hormone concentrations in the eggs of domestic chickens. We therefore experimentally elevated concentrations of maternal plasma corticosterone by implanting adult laying hens with corticosterone-releasing pellets and quantified gonadal hormones in the plasma of the hens and in the yolk of their eggs. First we performed a pilot experiment to establish the dose for treatment. Our results demonstrate that elevating maternal plasma corticosterone levels decreases reproductive hormone concentrations in the yolk.

## Materials and Methods

### Ethics Statement

This project was approved by the Institutional Animal Care and Use Committee of the University of Groningen (Permit Number: DEC 5124).

### Pilot study

#### Methods and results

**Figure 1:**
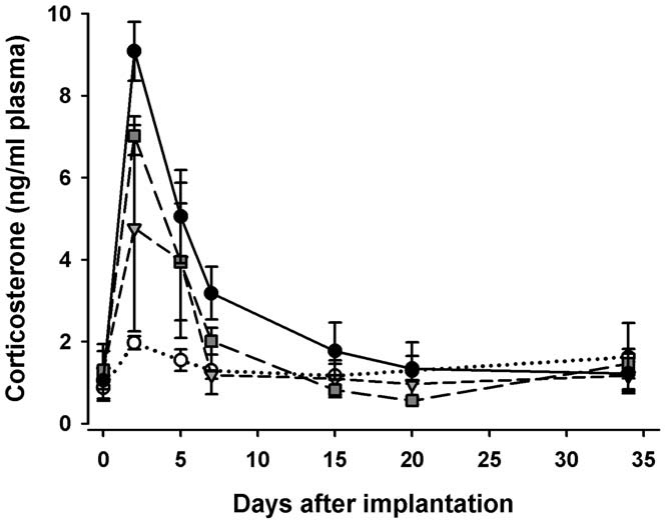
Corticosterone concentrations in plasma after pellet implantation. Mean (± SE) concentrations of corticosterone in plasma of each three hens implanted with 90-day corticosterone release pellets in four different pellet dosages (7.5 mg ○, 15 mg ▾, 20 mg ▪ and 30 mg •).

We wanted to obtain yolks that were formed while plasma corticosterone concentrations were constantly elevated. Once recruited into the follicular hierarchy, chicken follicles undergo a rapid growth phase during the final 6–11 days before ovulation [24], [25]. Our aim was therefore to elevate maternal plasma corticosterone concentrations for at least 10 days. We initially tested four different dosages of corticosterone-releasing pellets on three laying hens each. The pellets, obtained from Innovative Research of America (Sarasota, FL, USA), were designed for 60 days of continuous release of a total of 5 mg, 15 mg, 20 mg or 30 mg corticosterone, respectively. Chickens (for details see below) were caught and blood-sampled within 30 seconds to obtain baseline plasma levels of corticosterone. After disinfection of the skin in the flank region with alcohol and local lidocain application (Xylocain® Pumpspray, AstraZeneca, London, UK), a small incision was made into the skin with a scalpel. Using a blunt probe we created a subcutaneous pocket into which the pellet was inserted and closed the incision with one or two stitches afterwards. To assess the effectiveness of the corticosterone implants, hens were blood-sampled on days 2, 5, 7, 15, 20 and 34 after implantation. Blood samples were analysed for corticosterone as described below. We found a dose dependent transient peak in plasma corticosterone levels during the first days after implantation (Fig. 1). We chose to implant our chickens with the pellets of the 30 mg dosage in the main experiment as only this dosage elevated plasma corticosterone for the time necessary for egg formation. Hens implanted with this dose did not stop egg laying.

### Main experiment

#### Experimental animals

We used 20 white Leghorn and 20 ISA brown females aged 33 weeks and 10 sexually mature Rhode Island Red males in our main experiment. Animals were housed outdoors in May and June in ten aviaries (1.5 m×3 m), each of which contained two females from each strain and one male. Aviaries were separated from one another by wooden partitions. Commercial layer feed and water were provided ad libitum. Each aviary contained a laying nest, which was used by all four females. ISA brown hens are on average heavier than white Leghorns, but females were distributed such that in each aviary one white Leghorn hen was heavier than one ISA brown hen. Average body mass (mean±SEM) prior to implantation was 1.76±0.04 kg in ISA brown hens and 1.55±0.02 kg in white Leghorns, respectively. Five weeks before the implantation, rank order was assessed as described by Müller et al. [26]. In all aviaries, the two white Leghorn females were dominant over the two ISA brown hens and the heavier ISA brown hen was always dominant over the lighter hen. In only four out of ten aviaries was the heavier white hen dominant over the lighter white hen.

#### Treatment, blood sampling, egg collection and sample sizes

All hens were implanted with pellets as described above. Half of the females received 30 mg corticosterone pellets and the other half placebo pellets as control. The distribution of the pellets was balanced over rank and strain. Blood samples of about 0.5 ml were collected from the branchial vein on the day of implantation (day 0) and on days 1, 3, 6, 9 and 12 after implantation. Plasma samples were taken between 9 a.m. and 6 p.m. on the day of the implantation (day 0). After implantation, birds were blood sampled on the respective days between 12 a.m. and 3 p.m. in a random order.

The egg laying rate for each hen was assessed from 13 days before until 21 days after implantation. Mean (±SEM) number of eggs laid per day per individual prior to implantation was 0.88 (±0.02) for white Leghorns and 0.91 (±0.03) for ISA brown hens. To identify the eggs from the four individual females within each aviary we used powdered food colours that were administered into the cloacae of the hens on every other day, starting three weeks before the treatment. After the pellet-implantation, food colour was applied on the same days as the blood samples were drawn. As this method leaves subtle colour spots on the shell, every egg could be assigned to the correct female during the sampling period. Eggs were collected daily from day 0 (day of implantation) until day 19 after implantation. The collected eggs were kept at 4°C for four days at maximum until they arrived at the lab. There they were stored at -20°C until analysis for hormone concentrations was performed as described below. Eggs from days 12, 13, 15, 16 and 18 were incubated and hatched to assess the effects of maternal stress on the offspring (Henriksen et al., *in prep*).

The treatment failed in two of the corticosterone pellet-implanted ISA brown hens, i.e. their plasma corticosterone concentrations did not increase and the area under the curve was smaller than the highest values found in the placebo-implanted group. All eggs and plasma samples collected from these two females were therefore excluded from further analysis. The sample size for the blood samples in the hierarchical models (see below) was therefore ten females from each strain in the placebo pellet-implanted group and eight ISA brown and ten white Leghorn females in the corticosterone pellet-implanted group. After the implantation surgery, four hens stopped laying (two of the placebo pellet-implanted white Leghorns and one hen of each strain with corticosterone implants). The numbers of females in the yolk hormone and egg parameter analyses were therefore ten ISA browns with placebo pellets (135 eggs), seven ISA brown with corticosterone pellets (92 eggs), eight placebo pellet-implanted white Leghorns (98 eggs) and nine corticosterone pellet-implanted white Leghorns (82 eggs), respectively. In total, 407 eggs were collected and analysed. The number of eggs laid per day ranged from 22 to 34 since not all females produced eggs on all days.

#### Steroid hormone analysis

Plasma samples of 0.1 ml were extracted with diethyl ether, resuspended in 300 µl assay buffer and analysed via enzyme immunoassay (EIA) for corticosterone (antibody against corticosterone-3-CMO; [27]), testosterone (antibody against testosterone-3-CMO; [28]), progesterone (antibody against 5β-pregnane-3α-ol-20-one-3-HS; [29]) and estrogens (antibody against 17β-estradiol-17-HS; [28]). Sensitivities of the assays were 3 pg/well for the corticosterone EIA, 0.3 pg/well for the testosterone EIA, 0.1 pg/well for the estrogen EIA and 1.7 pg/well for the progesterone EIA. Intra- and inter-assay CV were 12% and 24%, respectively for the corticosterone EIA, 9% und 16% for the testosterone EIA, 20% and 25% for the progesterone EIA and 12% and 15% for the estrogen EIA.

The eggs collected for hormone analysis were weighed and defrosted until the albumin could be scraped off from the yolk with a spatula and the yolks were weighed. Concentrations of gonadal hormones in the yolk were measured with the EIAs mentioned above. As concentrations vary between the different regions of the yolk [8], [9], [30], we prepared three layers from each yolk to assess the hormone concentrations in the exterior, intermediate and interior regions of the yolk sphere. Samples were extracted by mixing 0.15 g of yolk with 0.6 ml of double-distilled water, homogenizing with a hand vortex and freezing overnight. On the next day, samples were shaken for 15 min before 3 ml of methanol were added. The mixture was shaken for 30 min and centrifuged at 2500 g for 15 min. For analysis with the progesterone EIA, the methanolic suspensions were simply diluted 1∶100 with assay buffer. For analysis with the testosterone and the estrogen EIA, 1 ml of the supernatant was transferred into a new vial. Eprouvettes were placed in a water bath at 60 C and the methanol evaporated under a stream of nitrogen. Samples were then resuspended in 0.5 ml of assay buffer. The minimum detectable hormone level was 0.29 ng per gram yolk for the testosterone EIA, 100 ng/g for the progesterone EIA and 0.07 ng/g for the estrogen EIA.

Hormone concentrations of whole yolks were calculated by multiplying the measured concentrations of each layer with the calculated weight of the respective layer, as described by Rettenbacher et al. [31], thereby considering that the different layers would contribute different amounts of yolk if homogenized prior to analysis.

Avian follicle cells have the enzymatic capacity to synthesize a plethora of steroid hormones and hormone metabolites [32] and it is therefore likely that yolk contains various steroids that have not yet been identified. The measured signal in the yolk might thus in fact be caused by two or more hormones or hormone metabolites that bind to the respective antibodies. We will refer to immunoreactive substances detected by our testosterone antibody as “yolk testosterone”, this term however including also other androgens and androgen metabolites that have a 17β-hydroxy structure, likewise we use “yolk progesterone” as characterized by Rettenbacher et al. [33] for substances measured with the progesterone antibody and “yolk estrogens”, comprising unconjugated estrogens measured with the estrogen antibody.

#### Statistical analysis

Data were analysed using the statistical software packages Statistica (StatSoft Inc., Tulsa, OK), SigmaStat (Systat Software Inc., San Jose, CA) and MlwiN (Version1.10.0007; [34]), the latter being able to accommodate hierarchical models to deal with a nested structure of unbalanced data. Two hierarchy levels were chosen for plasma hormones, yolk hormones and egg mass (samples within females within aviaries) and three levels for the hormone data of the different yolk layers (layers within egg within females within aviaries). Concentrations of plasma corticosterone and yolk testosterone were log transformed, all other plasma hormones and yolk progesterone were square-root transformed to meet criteria of normal distribution. In all models we tested the effects of treatment, time, strain as well as treatment*strain and treatment*time interactions. If the treatment*strain interaction was significant, each strain was tested post-hoc in a separate model, otherwise the interaction was removed from the model. Non-significant treatment*time interactions were removed from the final models, to assess a possible overall treatment effect. Results with an alpha <0.05 (two tailed t-test) were regarded as significant. Treatment effects within layers were also assessed in separate models for each layer. Yolk progesterone concentrations of the individual layers were not tested in a hierarchical model, as a normal distribution could not be achieved. Comparisons of the individual layers between treatment groups were therefore made via Mann-Whitney U-tests. Data are shown as means ± standard error of the mean (SEM). Plasma hormone concentrations on the individual days were also compared between the two treatment groups by using t-tests. Changes in laying performance before and after the implantation were calculated for each hen and treatments and strains were compared via ANOVA.

## Results

### Plasma hormone concentrations

**Figure 2:**
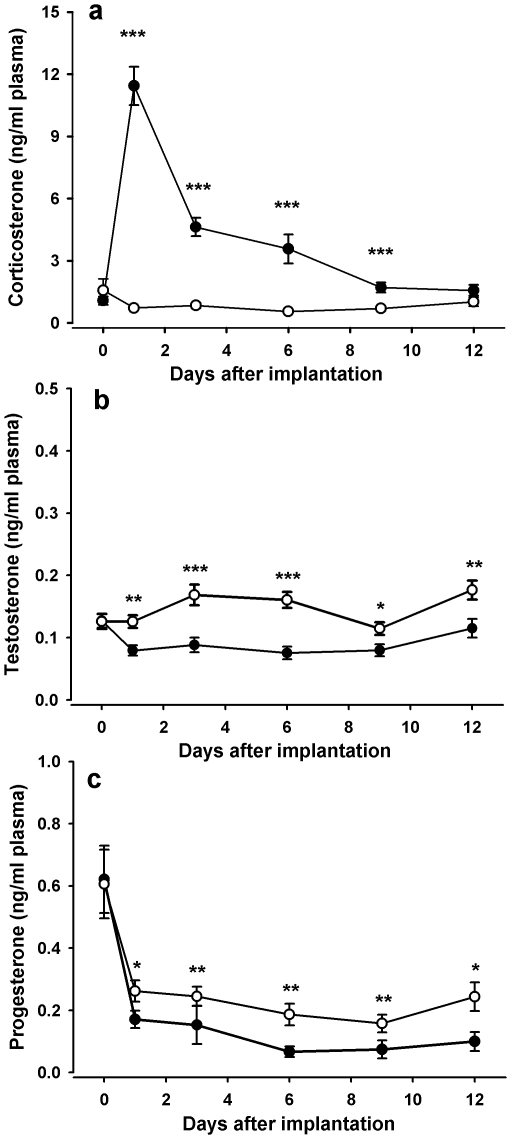
Hormone concentrations in plasma after pellet implantation. Mean (±SE) concentrations of corticosterone (a), testosterone (b), progesterone (c) in plasma of hens implanted with corticosterone (•) or placebo (○) pellets. Asterisks indicate significant differences between treatments on individual days (_***_ p≤0.001; _**_ p≤0.01; _*_ p≤0.05; t-tests).

**Figure 3:**
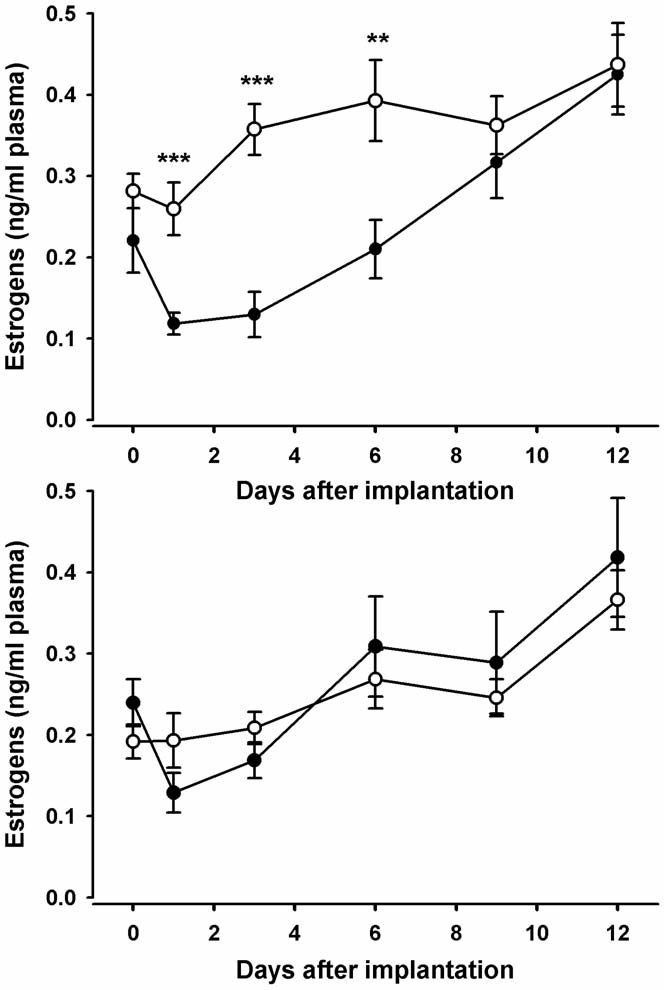
Estrogen concentrations in plasma after pellet implantation. Mean (±SE) concentrations of estrogens in plasma of white Leghorns (a) and ISA browns (b), after implantation with corticosterone (•) or placebo (○) pellets. Asterisks indicate significant differences between treatments on individual days (_***_ p≤0.001; _**_ p≤0.01; _*_ p≤0.05; t-tests).

Concentrations of plasma corticosterone were significantly elevated by treatment (treatment*time interaction: χ^2^ = 7.3; df = 1; p = 0.007), reaching peak concentrations during the first days after implantation, after which they gradually decreased (Fig. 2a). Treatment differences between strains were not detected (treatment*strain: χ^2^ = 0.02; df = 1; p = 0.89 and removed from the model). When treatments were compared on individual days, plasma corticosterone concentrations of the corticosterone pellet-implanted hens were significantly higher from day one until day nine after implantation (day 0 p = 0.99; day 1, 3, 6 and 9 p<0.001; day 12 p =  0.07). Concentrations of plasma testosterone were significantly decreased by the corticosterone pellet implantation, independent of strain and time (treatment: χ^2^ = 57; df = 1; p<0.001; treatment*strain χ^2^ = 3.38; df = 1; p = 0.066 and removed from the model; all other terms and interactions p≥0.17), from day one onwards during the entire sampling period (Fig. 2b; day 0 p =  0.9; day one p = 0.001; days three and six p<0.001; day nine p = 0.02; day 12 p = 0.007). Concentrations of plasma progesterone were also significantly decreased by treatment (treatment*time χ^2^ = 3.88; df = 1; p = 0.049) from day one onwards during the entire sampling period (Fig. 2c; day 0 p = 0.6; day one p = 0.048; days three and six p = 0.005; day nine p =  0.004; day twelve p = 0.03). Differences between strains were not detected (treatment*strain χ^2^ = 0.51; df = 1; p = 0.475 and removed from the model; strain χ^2^ = 0.69; df = 1; p = 0.4). Plasma estrogens were affected only in the white Leghorn strain (Fig 3a; treatment*strain: χ^2^ =  21.79; df = 1; p<0.001), where treatment decreased estrogen concentrations in the corticosterone pellet-implanted white Leghorn hens on day one (p<0.001), three (p<0.001) and six (p = 0.008) after implantation (post-hoc test: treatment*time: χ^2^ = 4.93; df = 1; p = 0.026). This effect was not found in hens of the ISA brown strain (Fig 3b; post-hoc test: treatment*time: χ^2^ = 1.16; df = 1; p = 0.28).

### Yolk hormone concentrations

**Figure 4:**
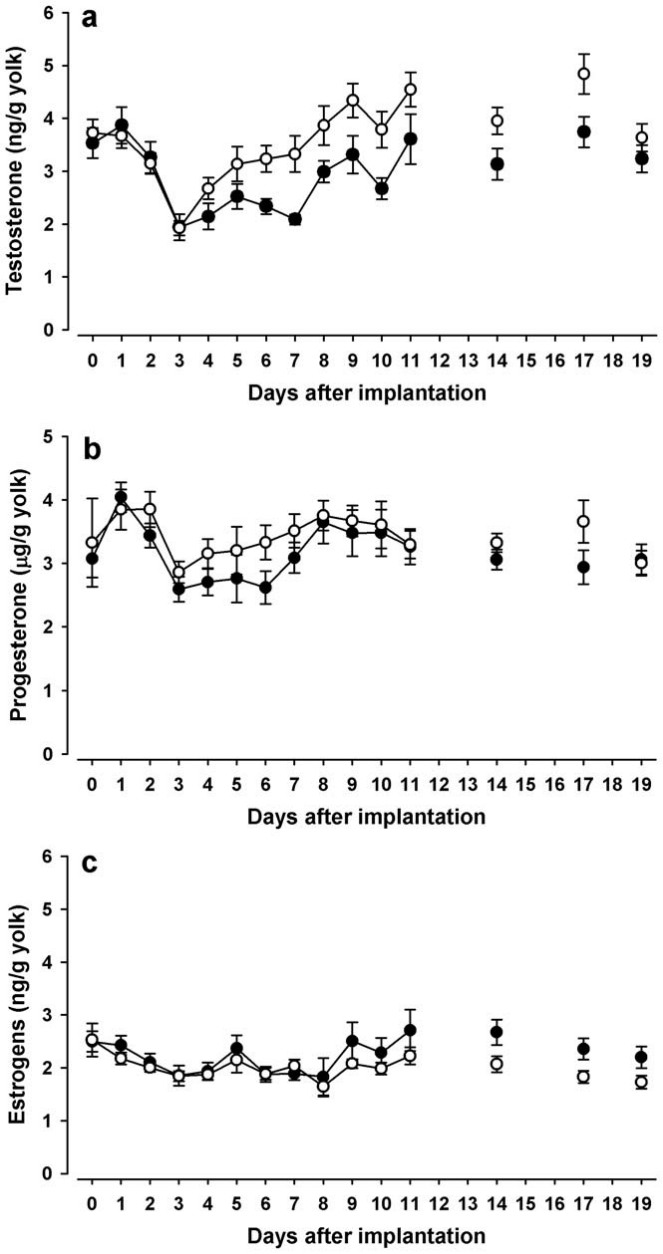
Hormone concentrations in yolk after pellet implantation. Mean (±SE) concentrations of testosterone (a), progesterone (b) and estrogens (c) in yolks from hens implanted with with corticosterone (•) or placebo (○) pellets. Eggs from days 12, 13, 15, 16 and 18 were incubated to hatch chicks. Note that progesterone concentrations are a thousand-fold higher than the other two steroid hormones.

Implanting hens with corticosterone pellets decreased the concentrations of yolk testosterone (Fig 4a; treatment*time χ^2^ = 6.56; df = 1; p = 0.01; treatment*strain χ^2^ = 2.51; df = 1; p = 0.11 and removed from the model). Mean (±SEM) yolk testosterone concentrations of all analysed eggs laid after implantation were 3±0.1 ng/g yolk in eggs from the corticosterone-implanted and 3.6±0.1 ng/g yolk for the placebo-implanted hens, respectively. Similarly, levels of yolk progesterone were also lower in eggs of the corticosterone-treated hens, with mean progesterone concentrations of 3156±74 ng/g yolk compared to 3430±78 ng/g yolk in controls (Fig 4b; treatment: χ^2^ = 5.5; df = 1; p = 0.019; treatment*time and treatment*strain not significant and removed from the model; p≥0.40). Yolk estrogens were not significantly affected by the treatment (Fig. 4c; treatment: χ^2^ = 15; df = 1; p = 0.7; treatment*time: χ^2^ = 0.65; df = 1; p = 0.42 and treatment*strain: χ^2^ = 2.2; df = 1; p = 0.14 and removed from the final model) with mean yolk estrogen concentrations of eggs from corticosterone- and placebo-implanted hens being 2.3±0.1 ng/g yolk and 2.0±0 ng/g yolk, respectively. Independent of treatment, ISA brown hens had significantly higher concentrations of yolk steroid hormones compared to white Leghorns. Mean concentrations of yolk testosterone (strain: χ^2^ = 31.6; df = 1; p<0.001) for corticosterone-implanted females were 3.3±0.1 ng/g and 2.7±1 ng/g for ISA browns and white Leghorns and 4±0.1 ng/g and 3.1±0.1 ng/g for placebo-implanted ISA browns and white Leghorns, respectively. Yolk progesterone concentrations were 3316±99 ng/g and 2975±106 ng/g for corticosterone-implanted and 3654±107 ng/g and 3127±106 ng/g for placebo-implanted ISA browns and white Leghorns, respectively (strain: χ^2^ = 13.3; df = 1; p<0.001) and yolk estrogen concentrations were 2.5±0.1 ng/g and 1.9±0.1 ng/g for corticosterone- and 2.1±0.1 ng/g and 1.9±0 ng/g for placebo-implanted ISA browns and white Leghorns, respectively (strain: χ^2^ = 6.8; df = 1; p = 0.009).

As treatment affected hormone concentrations differently in the three yolk layers (treatment*layer χ^2^ = 8.51; df = 2; p = 0.014), we tested hormone concentrations in the yolk layers in separate models. Implanting corticosterone-releasing pellets decreased yolk testosterone in the outermost layer (treatment: χ^2^ = 30.1; df = 1; p<0.001) with mean concentrations of 3.0±0.1 ng/g yolk and 3.7±0.1 ng/g in yolks from corticosterone-treated and control females and a similar tendency for the concentration in the inner layer of the yolks to decrease (treatment: χ^2^ = 3.7; df = 1; p = 0.052; mean concentrations 2±0.1 ng/g and 1.8±0.1 ng/g). Yolk testosterone concentrations in the middle layer were also lower in the corticosterone implanted females, but this difference did not reach statistical significance (3.1±0.1 ng/g and 3.6±0.1 ng/g in yolks of corticosterone- and placebo pellet-implanted females respectively; treatment: χ^2^ = 2.7; df = 1; p = 0.099; treatment*time: p≥0.08 for all layers in the respective models). Comparisons of the individual layers between treatment groups via Mann-Whitney U-tests did not reveal significant differences between yolk progesterone concentrations (p≥0.11 in all tests). We found mean concentrations of 4014±116 ng/g and 4221±103 ng/g for the outer, 1415±76 ng/g and 1444±79 ng/g for the middle and 319±40 ng/g and 311±28 ng/g yolk for the inner layers of the yolk in corticosterone pellet- and placebo pellet-implanted females, respectively.

### Egg parameters

Egg mass was lower in corticosterone-implanted hens (treatment: χ^2^ = 58.36; df = 1; p<0.001; treatment*strain: χ^2^ = 0.18 df = 1; p = 0.67 and treatment*time: χ^2^ = 2.69; df = 1; p = 0.1 and removed from the model) and varied significantly over time independent from treatment (time: χ^2^ = 4.02; df = 1; p = 0.04) whereas differences between strains were not detected (strain: χ^2^ = 0.18; df = 1; p = 0.67). Mean egg mass was 57.6±0.4 g for corticosterone pellet-implanted hens and 61.3±0.3 g for eggs from hens implanted with placebo pellets.

**Figure 5:**
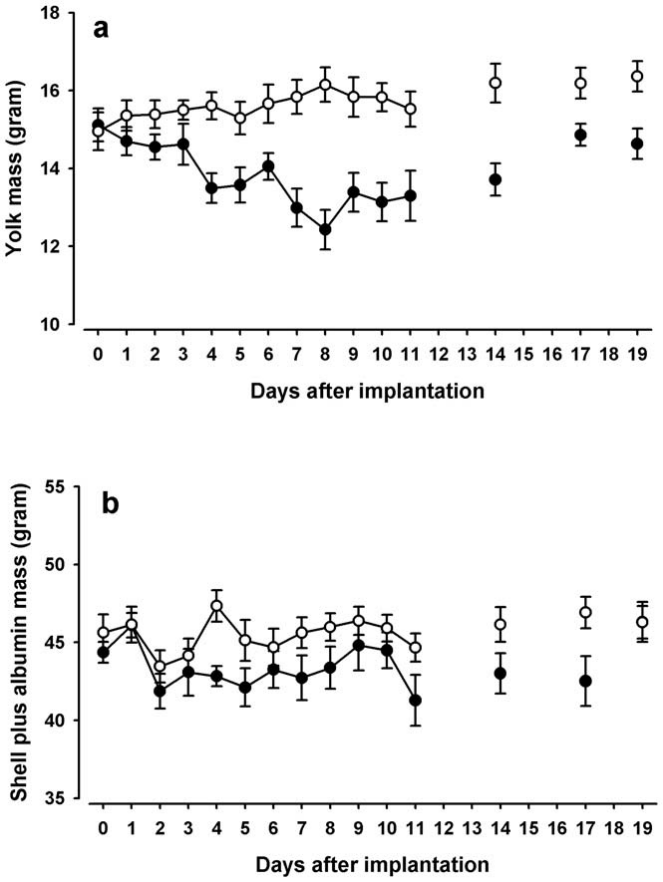
Egg mass after pellet implantation. Mean (±SE) yolk mass (a) and shell plus albumin mass (b) in eggs from hens implanted with corticosterone (•) or placebo (○) pellets. Eggs from days 12, 13, 15, 16 and 18 were incubated to hatch chicks.

Yolk mass was significantly lower in eggs from corticosterone pellet-implanted hens (Fig. 5a; treatment*time: χ^2^ = 6.87; df = 1; p = 0.009) and affected the two strains differently (treatment*strain: χ^2^ = 4.64; df = 1; p = 0.031) with the yolk mass decrease being more pronounced in ISA brown hens than in the white Leghorns (post-hoc tests for ISA browns and white Leghorns: treatment*time: χ^2^ = 6; df = 1; p = 0.01 and χ^2^ = 4.7; df = 1; p = 0.03, respectively). Yolks of corticosterone-treated ISA brown females had an average mass of 13.9±0.2 g, compared to 15.8±0.2 g in controls. In white Leghorns, mean yolk mass was 14.3±0.2 g and 15.5±0.1 g for corticosterone- and placebo pellet-implanted females, respectively. To assess egg mass differences that are not explained by the decrease in yolk mass, we subtracted yolk mass from egg mass to derive an estimate of the mass of both shell plus albumin together. Mean shell plus albumin mass was 43.5±0.3 g in eggs from corticosterone-implanted hens and this was significantly lower than in hens implanted with placebos, which laid eggs with a mean shell plus albumin mass of 45.6±0.3 g (Fig 5b; treatment: χ^2^ = 22.86; df = 1; p<0.001; treatment*strain χ^2^ = 1.9; df = 1; p = 0.16 and treatment*time χ^2^ = 0.63; df = 1; 0.426 and removed from the model).

### Treatment effect on egg laying

Egg laying decreased significantly in corticosterone pellet-implanted white Leghorn females only (F_(3,34)_ = 4.42; p = 0.03). Mean (±SEM) number of eggs laid between day 0 and day 19 after implantation was 12±1.9 for white Leghorns with corticosterone pellets, 18±0.48 for ISA browns with corticosterone pellets and 17.5±0.6 and 18.5±0.54 for white Leghorns and ISA browns with placebo implants, respectively.

## Discussion

Despite extensive research on hormone-mediated maternal effects in birds, the physiological mechanism by which a female bird deposits different amounts of gonadal hormones into her eggs is still unresolved. The present study investigated the potential effect of corticosterone on gonadal hormones in maternal plasma and egg yolk. Our findings indicate that elevating plasma corticosterone concentrations suppressed gonadal hormone synthesis in the ovarian follicles, which was reflected in decreased concentrations of reproductive hormones in both plasma and yolk.

### Effectiveness of the corticosterone implants

Implanting laying hens with corticosterone-releasing pellets designed for 60 days of continuous corticosterone release elevated plasma corticosterone concentrations for only nine days post implantation with a pronounced peak during the first three days. This is in accordance with observations made by Bonier et al. [35] and Müller et al. [36], who used pellets of the same provider in other bird species and also found much shorter elevations than indicated by the manufacturer. From this we conclude that these pellets elevate plasma corticosterone concentrations in birds much shorter than in mammals. This might be due to an increase in metabolic clearance rate by enhanced enzymatic activity, faster breakdown of the biodegradable matrix of the pellet and/or a downregulation of binding globulins and should be investigated in more detail.

Deposition of radiolabelled hormones or substances such as Sudan dyes into the yolk of chickens occurs during the last 11 days of yolk formation [25], [31], [37] and an additional 25-hour period is necessary for albumin and shell formation [38]. Therefore in the present study, all eggs collected until day 19 after implantation were therefore at least partly yolked when the females' plasma corticosterone levels were elevated.

### Effects on reproductive steroid hormones

Yolks produced by corticosterone pellet-implanted females contained less yolk testosterone and progesterone than yolks from placebo pellet-implanted females.

Females implanted with corticosterone pellets also had lower plasma progesterone and testosterone concentrations. This is consistent with the fact that GC are generally considered hormones with antigonadotropic action, as they shift physiological functions away from reproduction towards survival, especially under long-term conditions [20]. Stressful conditions such as high temperatures have also been shown to down-regulate the plasma concentrations of reproductive hormones in chickens [39]. In accordance with our results, Okuliarova et al. [40] found lower testosterone concentrations in the follicles of Japanese quails under severe restraint stress. A recent study in quails revealed that confrontation with humans also decreases testosterone and androstenedione but elevates yolk progesterone levels [11]. Housing quails in unstable groups however increased yolk testosterone concentrations, whereas yolk progesterone and androstenedione were unaffected [12]. These inconsistent findings suggest that different types of stressors exert different effects on yolk steroid hormones and illustrate that further research is necessary. Despite its presence in high concentrations, yolk progesterone has not received much attention in the past and its role in the development of the bird embryo is unknown. The finding that progesterone and other gestagen metabolites affect the growth of chick embryo cartilage [41] however suggests a possible role for these hormones in the development of bird embryos. In mammals, prenatal progesterone is known to enhance cognitive ability [42].

Gonadal hormones are produced by the theca and granulosa cells of the ovarian follicle [43]. During the course of maturation, the steroidogenic activity of a follicle changes due to different responsiveness to LH and FSH stimuli [44]. When we analyzed steroid hormone concentrations of the different yolk layers separately, we found the most pronounced decrease of yolk testosterone in the outer layers of the yolk, which are produced at a later stage. It might therefore be possible, that the corticosterone treatment affected the individual follicles differently, depending on their actual maturation stage. Our findings suggest that larger follicles were more affected, whereas small follicles are less susceptible. As estrogens are synthesized exclusively by the early stage follicles [45], this would also explain why estrogens were least affected by our treatment. In white Leghorns, plasma estrogens were only suppressed for three days, whereas progesterone and testosterone concentrations were lower during the entire sampling period. This is in accordance with findings in white Leghorns [39], where heat stress affected concentrations of plasma estrogens less than plasma testosterone or progesterone and suggests that the smaller follicles either recover faster and resume their estrogen production or that they are quickly replaced by new re-growing follicles.

One might wonder why estrogen concentrations decreased in the plasma but not yolks of white Leghorns. Although there are still many open questions regarding the relationship between plasma and yolk hormone concentrations and more knowledge about partitioning mechanisms of steroid hormones between blood and egg is therefore certainly necessary [15], we believe we have identified several reasons why linking plasma and yolk hormone concentrations at the individual level might be hampered: First, yolk hormone levels reflect hormone accumulation within an individual follicle over a certain period of time, thereby integrating hormone production over several days, whereas plasma hormones represent a single time point measurement. Also, plasma concentrations can fluctuate due to changes in blood volume, enhanced clearance from blood via enzymatic degradation, changes in binding proteins, etc., whereas potential influences on the amount of hormones that are transferred into the yolk are still largely unknown. Furthermore, plasma concentrations represent a pool into which all active follicles contribute, whereas yolk hormones are mostly produced by the cells surrounding the individual follicles [14], [15]. The latter is supported by the fact that yolk steroid hormones vary among yolk layers, reflecting both amount and type of hormones produced during the maturation of the individual follicle. In addition, yolk contains a mixture of several different hormones or hormone metabolites [33]. Therefore, the measured concentration in the yolk might be caused by several immunoreactive substances which bind to the antibody. If those hormones are not present in similar proportions in the plasma, a potential correlation between plasma and yolk hormone concentrations cannot be detected.

Moreover, we observed no difference in plasma hormone levels between the two strains, whereas concentrations of yolk testosterone, estrogens and progesterone were higher in eggs from ISA brown hens than in white Leghorns, irrespective of treatment. To our knowledge, differences in concentrations of yolk steroid hormones have not been assessed previously between layer hen strains. Yolk hormone concentrations in quails are highly heritable [46], [47], suggesting a genetic component to variations. The observed differences in yolk steroid hormone concentrations between the two chicken strains could be due to either differences in hormones production, or because different amounts of hormones are transferred actively or passively into the yolks. For the latter, strain differences in yolk composition might also be relevant [48].

### Effects on egg parameters and egg laying

Our treatment decreased laying performance only in white Leghorns, whereas yolk mass reduction was seen in both strains although more pronounced in the ISA brown hens. Implantation with corticosterone pellets also decreased shell and albumin mass in both strains. Corticosterone treatment has been shown to reduce the production of yolk precursors in the liver [49], to decrease egg mass [50] and to suppress laying performance [21], [51]. These effects could be due to the down-regulation of gonadal hormones, which are essential for reproduction [24], but may also be explained by the fact that glucocorticoids influence both protein and fat metabolism in birds [52], [53] and thus yolk formation. Our treatment decreased concentrations of yolk testosterone and progesterone as well as yolk mass, thus resulting in an overall reduction of total hormone content in the eggs. Smaller yolks would also limit the amount of nutrients available for the chick embryo. In mammals, fetal malnutrition is one of the main mediators of maternal stress, because GC can compromise placental capacity for nutrient transfer [54]. As the exact modes of action of maternally derived hormones on the bird embryo have so far been described only partially [55], it remains largely unknown by which mechanisms the altered composition and amounts of egg components affect the developing offspring.

Whether corticosterone itself was transferred to the eggs due to the treatment was not assessed in the present study. Hormone transfer from plasma to yolk is generally low [30], [31], but high concentrations of plasma corticosterone, e.g. after feeding 0.1 g of crystallized corticosterone were reflected in elevated yolk corticosterone concentrations [31]. It has to be noted however, that quantification of corticosterone in the yolk is not a straightforward procedure: As we showed recently, the high concentrations of gestagens present in egg yolk of chickens interfer with the quantification of corticosterone via immunoassays [33]. To assess whether quantification of yolk corticosterone is possible via other analytical methods was beyond the scope of this study. In the present study we investigated a possible physiological mechanism responsible for varying concentrations of reproductive hormones in bird eggs. Our current findings indicate that corticosterone from the mothers' circulation can modulate the concentrations of testosterone and progesterone in the yolk of domestic chickens. They also suggest that maternal stress affects the bird embryo indirectly via reproductive hormones and nutrient amount. As we artificially elevated plasma corticosterone levels, further investigations are necessary to clarify whether a more natural stressor triggers similar physiological responses in egg-laying females and whether wild bird species respond in the same way as domestic chickens.
